# A precision medicine approach to a patient with unresolved pain following orthopedic surgery: a case report

**DOI:** 10.1186/s13256-017-1207-5

**Published:** 2017-02-24

**Authors:** David Gazzaniga, Ashley Brenton, Brian Meshkin

**Affiliations:** 1 Newport Orthopedic Institute, Newport Beach, CA USA; 2 Proove Biosciences, 15326 Alton Pkwy, Irvine, CA 92618 USA

**Keywords:** Precision medicine, Pain management, Orthopedics, Predictive algorithms, Opioid risk

## Abstract

**Background:**

Precision medicine is a promising technology in patient care that combines genetic analysis with clinical data, such as health, behavioral, functional, environment, and lifestyle information. Here we present the case of a 54-year old woman who, following an accident, had uncontrolled chronic pain and was subsequently labeled a drug seeker.

**Case presentation:**

A 54-year-old white woman who was experiencing severe calf pain was referred for treatment. Her pain was insufficiently controlled immediately following knee arthroplasty with multiple opioid medications, as well as non-opioids. Precision medicine testing was ordered for her so that we could assess her pain sensitivity objectively to determine if the pill seeker designation was correct and to determine the best medications for her.

Based on the Proove profiles, we determined that she had moderately low pain sensitivity, which means that clinically she may underreport pain and may have decreased medication needs. This result suggested that her continued reporting of unresolved pain was probably due to a condition unresolved by her right knee arthroplasty. In addition, she was found to be at low risk of opioid addiction, based on the Proove Opioid Risk Profile. Taken together, along with the high levels of pain she described, we determined that her pain was not properly controlled and that the designation of pill seeker was incorrect. The next step was to determine which medications and which doses would result in the most favorable outcomes for our patient. To determine this, we used the results of the Proove Opioid Response, Proove Drug Metabolism, and Proove Non-Opioid Profiles to guide her treatment. We reduced her pain medications to a single opioid, Vicodin (acetaminophen and hydrocodone), which also eliminated the adverse side effects she experienced.

**Conclusions:**

Precision medicine offers an important health care decision tool which can reduce emotional and physical costs to patients and may reduce the economic health care burden of unnecessary surgeries and ineffective medication. The information provided by these profiles can be used clinically to guide treatment decisions and evaluate patient pain.

## Background

Precision medicine is a promising technology in patient care that combines genetic analysis with clinical data, such as health, behavioral, functional, environment, and lifestyle information. Here we present the case of a 54-year-old woman who, following an accident, had uncontrolled chronic pain and was subsequently labeled a drug seeker. Precision medicine testing revealed that she was at low risk of opioid use disorder, had a moderately low pain sensitivity score, and had a genotype that was incompatible with favorable response to her current medications. Following treatment changes guided by the results of the precision medicine profiles, her previously uncontrolled pain resolved and her function and quality of life improved.

## Case presentation

A 54-year-old white woman who was experiencing severe calf pain was referred for treatment. She had undergone a right knee arthroplasty following initial complaints of leg pain in December 2015. Following surgery, she experienced excruciating pain, as well as pain in the posterolateral aspect of her knee and some neurological sensation of numbness and weakness. The pain was insufficiently controlled immediately following surgery with OxyContin (oxycodone) 10 mg and two tablets of Percocet (acetaminophen and oxycodone) 10/325 mg in the morning, one to two Percocets (acetaminophen and oxycodone) at 3 p.m., one Percocet (acetaminophen and oxycodone) at 7 p.m., one to two Percocets (acetaminophen and oxycodone) at bedtime, as well as OxyContin (oxycodone), Norco (acetaminophen and hydrocodone), Vicodin (acetaminophen and hydrocodone), and MS Contin (morphine sulfate). To combat opioid-induced side effects, she was prescribed Movantik (naloxegol) for constipation and Zofran (ondansetron) for nausea. In addition to the listed medications, she was also prescribed Robaxin (methocarbamol), DILT-CD (diltiazem hydrochloride), fluticasone propionate, furosemide, tizanidine hydrogen chloride (HCL), and Xanax (alprazolam). She was unable to put any weight on her leg. The treating medical team felt that she was exaggerating her pain, which she rated as an 8 on a numerical rating 10-point scale and refused her requests for additional pain relief. She was discharged 3 days after surgery. Following her continued requests for pain relief medication, she was labeled a “drug seeker” and was referred to a pain management clinic. She states that she felt embarrassment and humiliation.

At the end of January 2016, she was examined by the first author, as a new treating physician. We ordered precision medicine testing from Proove Biosciences, Inc. (Irvine, CA, USA) to objectively gain insights into her: (1) pain sensitivity, (2) drug-metabolizing enzyme genotypes which may affect dose or adverse event profiles, (3) risk for opioid use disorder, and (4) likelihood of response to the most commonly prescribed opioid and non-opioid pain medications.

Based on the Proove profiles, we determined that she had moderately low pain sensitivity, which means that clinically she may underreport pain and may have decreased medication needs. This result suggested that her continued reporting of unresolved pain was probably due to a condition unresolved by the right knee arthroplasty. In addition, she was found to be at low risk of opioid addiction, based on the Proove Opioid Risk Profile. Taken together, along with the high levels of pain she described, we determined that her pain was not properly controlled and that the designation of pill seeker was incorrect. The next step was to determine which medications and which doses would result in the most favorable outcomes for our patient. To determine this, we used the results of the Proove Opioid Response, Proove Drug Metabolism, and Proove Non-Opioid Profiles to guide her treatment. We reduced her pain medications to a single opioid, Vicodin (acetaminophen and hydrocodone), which also eliminated the adverse side effects that she had experienced. This treatment, guided by the Proove profile, provided the necessary analgesia. However, the medication still did not resolve the underlying issue.

Beyond medication management, the precision medicine profile illustrated that she must have a remaining injury or musculoskeletal damage causing the uncontrolled pain even after her right knee arthroplasty. On further examination, we found an exostosis and heterotopic ossification of her popliteus muscle. This was surgically removed by a posterolateral approach to the proximal tibia. Following this surgery, her pain was substantially better than it was after her knee arthroplasty.

## Discussion

The Proove precision medicine profiles combine genetic risk factors with other clinical variables in proprietary algorithms to provide information on patient pain sensitivity, disease risk, medication response, and risk of substance use disorder.

Precision medicine provides physicians with a tool to optimize patient treatment, as well as providing objective information about a patient’s pain perception. Other measures of pain perception are generally subjective and self-reported by the patient, using a pain rating scale (Numeric Rating Scale or Visual Analog Scale) or through the Brief Pain Inventory [1] or McGill Pain Questionnaire [2, 3]. In contrast, objective information, such as that provided by the Proove Pain Perception Profile, provides physicians with evidence-based measures of pain perception to inform their treatment decisions.

Genetic testing is routinely used in cardiology [4], oncology [5], and hematology [6] and the field of pharmacogenetics is increasingly recognized as an important part of emerging precision medicine efforts [7]. In fact, the implications of genetic variation on medications used in pain management are well established in the literature [8, 9]. In this case, our patient’s medications prior to genetic testing were unable to control her pain and, following testing, a reduction in the number of medications and targeted treatment resulted in satisfactory analgesia for the first time since the initial injury, without the polypharmacy-related adverse drug events.

## Conclusions

Precision medicine offers an important health care decision tool which can reduce emotional and physical costs to patients and may reduce the economic health care burden of unnecessary surgeries and ineffective medication. The information provided by these profiles can be used clinically to guide treatment decisions and evaluate patient pain.
